# 3D Models Reveal the Influence of Achilles Subtendon Twist on Strain and Energy Storage

**DOI:** 10.3389/fbioe.2021.539135

**Published:** 2021-02-05

**Authors:** Katherine R. Knaus, Silvia S. Blemker

**Affiliations:** Department of Biomedical Engineering, University of Virginia, Charlottesville, VA, United States

**Keywords:** Achilles tendon, fascicle twist, tendon strain, tendon energy storage, subtendon morphology, finite element modeling, free tendon loading

## Abstract

The Achilles tendon (AT) has complex function in walking, exchanging energy due to loading by the triceps surae muscles. AT structure comprises three subtendons which exhibit variable twist among themselves and between individuals. Our goal was to create 3D finite element (FE) models to explore AT structure-function relationships. By simulating subtendon loading in FE models with different twisted geometries, we investigated how anatomical variation in twisted tendon geometry impacts fascicle lengths, strains, and energy storage. Three tendon FE models, built with elliptical cross sections based on average cadaver measurements, were divided into subtendons with varied geometric twist (low, medium, and high) and equal proportions. Tendon was modeled as transversely isotropic with fascicle directions defined using Laplacian flow simulations, producing fascicle twist. Prescribed forces, representing AT loading during walking, were applied to proximal subtendon ends, with distal ends fixed, and tuned to produce equal tendon elongation in each case, consistent with ultrasound measurements. Subtendon fascicle lengths were greater than free tendon lengths in all models by 1–3.2 mm, and were longer with greater subtendon twist with differences of 1.2–1.9 mm from low to high twist. Subtendon along-fiber strains were lower with greater twist with differences of 1.4–2.6%, and all were less than free tendon longitudinal strain by 2–5.5%. Energy stored in the AT was also lower with greater twist with differences of 1.8–2.4 J. With greater subtendon twist, similar elongation of the AT results in lower tissue strains and forces, so that longitudinal stiffness of the AT is effectively decreased, demonstrating how tendon structure influences mechanical behavior.

## Introduction

The Achilles tendon (AT) plays an important but complex role in human movement. This unique tendon transmits forces from the three muscles of the triceps surae to the calcaneus during production of ankle plantarflexion torque. Due to muscle loading, the AT stores and returns energy as it stretches and recoils (Lichtwark and Wilson, 2005; Zelik and Franz, 2017). Each of the triceps surae muscles perform different functions in the generation of propulsion and vertical support during walking (Neptune et al., 2001; Anderson and Pandy, 2003; McGowan et al., 2008; Francis et al., 2013), and each muscle has a unique architecture (Ward et al., 2009; Handsfield et al., 2014; Bolsterlee et al., 2019). Therefore, the three triceps surae muscles apply different forces to the AT (Arndt et al., 1998), which means that the AT functions as three combined tendons, leading to a more complex relationship between structure and function than in a tendon attached to a single muscle.

The evidence of complex loading conditions can be visualized using ultrasound imaging during walking. For example, non-uniform displacements have been observed in the *in vivo* free tendon; the deep portion of the tendon displaces more than the superficial portion between toe-off and mid-stance, indicating greater elongation of this region (Franz et al., 2015). These kinematic results likely occur due to the combination of complex loading and the complicated structure of the AT. The internal structure of the Achilles free tendon comprises three subtendons (Handsfield et al., 2016), which are distinguishable groups of fascicles that originate from individual muscles: the gastrocnemius lateral head, medial head, and the soleus. These subtendons have been observed in cadavers to twist around each other before inserting into the calcaneus (Cummins and Anson, 1946; Szaro et al., 2009; Edama et al., 2015). The twisted structure occurs in all ATs and the direction of twist is consistent across individuals. However, the amount of subtendon twist varies between individuals, such that previous authors have used that variation to classify tendons into three groups (Cummins and Anson, 1946; Edama et al., 2015; Pękala et al., 2017). These detailed anatomical studies present many questions about the functional consequences of AT internal structure and how mechanical behavior may vary with differences in anatomy. For example, would strain experienced by the tendon during a given elongation vary with differences in the twisted structure of its subtendons? Would differences in subtendon twist change the amount of energy stored during that same stretch?

Computational models enable exploration into the relationships between tendon structure and function. A finite element (FE) model of Achilles subtendons (Handsfield et al., 2017b) demonstrated how sliding and differential loading are possible mechanisms underlying observed non-uniform displacements (Slane and Thelen, 2015). Shim et al. (2018) developed subject-specific models of the Achilles free tendon in which they incorporated variations in fascicle twist. Their study confirmed that varying tendon twist does impact mechanical behavior. However, tendon geometry and material properties also varied in this study and have been shown to be highly variable between individuals and contribute to differences in mechanical behavior (Shim et al., 2014). It remains unclear to what extent observed fascicle twist within the subtendons may influence tendon mechanics, independent of these other variations in the AT.

Variation in subtendon twisted morphology could also affect *in vivo* measurements of tendon mechanical behavior. For example, strains in the AT are generally estimated by tracking the distance between the distal and proximal endpoints (Kubo et al., 2002; Lichtwark and Wilson, 2005; Onambele et al., 2006), assuming that strain is a linear measure between these two endpoints. Similar methods are employed in combination with force estimation to determine tendon work (Lichtwark and Wilson, 2005; Zelik and Franz, 2017). It is possible that tendon fibers twisted along the length of the tendon may affect the relationship between the tendon tissue strain and energy storage and the longitudinal estimates of strain and energy storage.

The goal of this work was to investigate how differences in subtendon internal twisted structure influences AT fascicle morphology, strains, and energy storage. Our secondary goal was to explore how subtendon twisting may affect quantification of AT strain and energy storage made with *in vivo* measurements. In order to quantify the effects of twist geometry, independent of differences in free tendon shape and material properties, we built a FE model of the AT with three different internal structures. The different versions of the model represented the average of the three classifications of Achilles subtendon fascicle twisting observed in unloaded cadaver tendons (Pękala et al., 2017) and simulations were performed in which the tendon models were loaded to represent tendon displacement in walking. We analyzed the simulations to (1) determine how functional tendon behavior varied with differences in subtendon twisted morphology, and (2) assess how varied morphology contributed to errors in quantification of strain and energy storage *in vivo*.

## Materials and Methods

### Model Geometries

**FIGURE 1:**
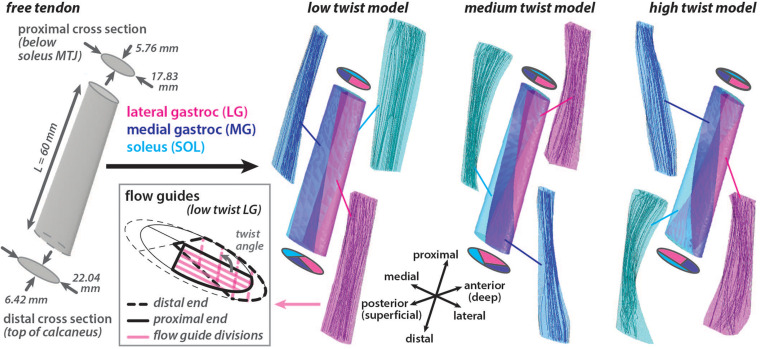
Achilles free tendon geometry was developed with elliptical cross sections at the ends. Three unique models with differing amounts of subtendon twist in the undeformed configuration were created by subdividing the free tendon geometry using internal surfaces. These surfaces had the same proximal subtendon cross section divisions and differing distal cross section divisions. Flow guides were used to direct the amount of fascicle twist in each subtendon by creating surfaces that were parallel in the axial cross sections that divided the subtendon volume. Flow guide surfaces are shown in the axial plane for the proximal and distal ends of the lateral gastrocnemius (LG) subtendon in the low twist model. The angle between the flow guides at the ends defined the *twist angle* of the fascicles within the subtendon. Fascicle tracts in the undeformed models, reconstructed from the resulting fiber directions, are shown.

A three dimensional (3D) Achilles free tendon geometry was created in Autodesk Inventor (Autodesk Inc. San Rafael, CA, United States) based on measurements from Pękala et al. (2017). Two elliptical axial cross sections were defined a distance of 60 mm apart and a surface was lofted between them to create a 3D geometry with a volume of 5804.85 mm^3^ (Figure 1). The proximal cross section (major axis = 17.83 mm, minor axis = 5.76 mm, area = 80.66 mm^2^) represented the point on the tendon that is just distal to the soleus musculotendinous junction (MTJ). This location was chosen as the most proximal location that the free tendon can be assumed to be in series with all three triceps surae muscles (Epstein et al., 2006). The distal cross section (major axis = 22.04 mm, minor axis = 6.42 mm, area = 111.13 mm^2^) represented the point on the tendon that is just proximal to the superior portion of the insertion into the calcaneus.

Using this 3D tendon geometry, three models were created with different internal structures characterized by the amount of subtendon twist. Surfaces divided each 3D model into three subtendons that corresponded with each of the triceps surae muscles: lateral gastrocnemius (LG), medial gastrocnemius (MG), and soleus (SOL). Subtendon divisions were defined such that the proximal cross sections were the same in each model while the distal cross sections varied, resulting in three unique undeformed models with differing sub-structure geometries: low twist, medium twist and high twist (Figure 1). The distribution of volume between the three subtendons was the same in all models (LG = 44%, MG = 27.5%, SOL = 28.5%). The three model geometries corresponded with the average of the classifications of AT torsion (Type I, Type II, and Type III) described by Pękala et al. (2017). These twisted model geometries were the undeformed or resting configuration of models where there was zero stress.

### Model Fascicles

Each model was meshed automatically into 3D tetrahedral elements (AMPS Technologies, Pittsburgh, PA, United States). The low twist model contained 4,336 nodes and 16,595 elements, the medium twist model contained 4,147 nodes and 16,168 elements, and the high twist model contained 4,291 nodes and 16,355 elements. Each subtendon was meshed independently so that nodes on adjacent surfaces were not shared. An element convergence analysis was performed by repeating simulations of the low twist model with different mesh densities. The selected mesh was chosen such that the differences in the primary metrics of average *along-fiber strain* was less than 0.1% and in total *strain energy* was less than 3% when the number of elements increased by a factor of ten. Additionally, the maximum and minimum strain differed by 5 and 3%, respectively, and the first principle stress differed by less than 3%.

A local fiber direction (*a*_0_) was defined for each element to represent the tendon fascicle structure, with a previously described method utilizing Laplacian simulations (Handsfield et al., 2017a). For each 3D subtendon, fibers were directed from the proximal cross section (inlet surface) to the distal cross section (outlet surface). When characterizing of three tendon twist types, Pękala et al. (2017) dissected the individual subtendons and quantified the average degree of twist of the fascicles in each unloaded subtendon. Based on these measurements, fascicle twist in each undeformed subtendon was enforced by implementing flow guides (Handsfield et al., 2017a) that were internal surfaces that divided the subtendons into four twisting portions. These surfaces were parallel in axial cross sections. The *twist angle* was defined as the angle in the axial plane between the proximal and distal edges of the flow guides (Figure 1). Unique flow guides resulted in different fascicle *twist angles* in each model (low twist model: LG = 107°, MG = 17°, SOL = 105°; medium twist model: LG = 157°, MG = 35°, SOL = 145°; high twist model: LG = 211°, MG = 68°, SOL = 200°).

To compute the lengths of the subtendon fascicles in the undeformed models, streamlines, generated at seed points on the proximal surface, were mapped through the field of local fiber direction vectors (*a*_0_) in MATLAB (MathWorks Inc., Natick, MA, United States). These streamlines defined fascicle tracts, which were then truncated to not extend beyond the volume of the subtendon geometry or extrapolated to terminate on the distal surface using a method adapted from Bolsterlee et al. (2017). We defined *fascicle lengths* as the lengths of the adjusted fascicle tracts as they twisted from the proximal origin to distal insertion were calculated as previously described (Bolsterlee et al., 2017). At least 250 fascicle tracts were created for each model subtendon, representing the internal fascicle geometry in the undeformed condition with zero strain.

### Constitutive Model

Subtendons were modeled as transversely isotropic, hyperelastic, quasi-incompressible material (Weiss et al., 1996; Criscione et al., 2001; Blemker et al., 2005). The constitutive model has been described in detail by Blemker et al. (2005) and has the strain energy density function defined in Eq. 1:

(1) $$\text{Φ}\,\left(C,{\text{a}}_{0}\right)=W_{1}+W_{2}+W_{3}+{\text{Φ}}^{v\,o\,l}$$

where **a**_0_is the local fiber direction, **C** is the right Cauchy-Green deformation tensor. The dilatational portion of the strain energy (${\text{Φ}}^{v\,o\,l}=\frac{K}{2}\,\text{ln}\,\left(J^{2}\right)$) relates to the volume change where $J=\sqrt{\text{det}\,\left(C\right)}$ and depends on a bulk modulus with a value set to *K* = 5e3 MPa. The strain energy associated with along-fiber shear ($W_{1}=G_{1}\,{\left(B_{1}\,\left({\bar{I}}_{4},{\bar{I}}_{5}\right)\right)}^{2}$) depends on a shear modulus set to *G_1_* = 3 MPa and the strain energy associated with cross-fiber shear ($W_{2}=G_{2}\,{\left(B_{2}\,\left({\bar{I}}_{1},{\bar{I}}_{4},{\bar{I}}_{5}\right)\right)}^{2}$) depends on a shear modulus set to *G_2_* = 15 MPa (Fiorentino and Blemker, 2014). ${\bar{I}}_{1},{\bar{I}}_{4},{\bar{I}}_{5}$ are deviatoric invariants of *C*. The function for the strain energy associated with along-fiber stretch (*W*_*3*_) characterizes the relationship between Cauchy stress in the tendon (σ) and the fiber stretch ($\lambda =\sqrt{{\bar{I}}_{4}}$) and is defined to be consistent with a piece-wise tendon stress-strain relationship in Eq. 2:

(2) $$\lambda \,\frac{\partial W_{3}}{\partial \lambda }=\left\{ \begin{array}{c} \sigma \,\left(\lambda \right)=P_{1}\,\left(e^{P_{2}\,\left(\lambda -1\right)}-1\right)\,1<\lambda <\lambda^{*}\\ \sigma \,\left(\lambda \right)=P_{3}\,\lambda +P_{4}\;\lambda \geq \lambda^{*}\; \end{array}\right.$$

where λ* represents the fiber stretch at which σ becomes linear and was set to λ* = 1.03. In the piece-wise equation, *P*_3_ and *P*_4_ were defined so σ is C^0^ and C^1^ continuous at λ=λ* *P*_1_ and *P*_2_ were set to values of 1.75 MPa and 48.3, respectively, so that the slope in the linear region was 360 MPa (Onambele et al., 2006).

The constitutive model was implemented in the multi-physics FE analysis program, AMPSol (AMPS Technologies, Pittsburgh, PA, United States), by creating a user-defined hyperelastic material with explicit strain energy function specification.

### Model Boundary Conditions

Frictionless sliding contact was assigned between the surfaces of adjacent subtendons in each model. The mechanics of the inter-subtendon matrix in the human AT are unknown and the interfascicular matrix has been shown to allow relative sliding of tendon fascicles in comparative studies (Thorpe et al., 2015), so this approach has been used previously to model Achilles subtendon interaction (Handsfield et al., 2017b). The distal end of each sub tendon was fixed, and the proximal end was constrained to move only in the proximal-distal direction.

To simulate uniaxial loading applied to the AT during walking, pressure boundaries were applied to the proximal surface of each subtendon. The applied pressure on each subtendon was tuned so that in each model the displacement of the proximal surfaces of the MG and LG subtendon were 7.6 mm and the displacement of the proximal SOL subtendon surface was 5.9 mm. These displacements were determined based on measurements of the maximum elongations measured in the superficial and deep portions of the AT during walking (Franz et al., 2015). These *in vivo* subtendon elongations were estimated from the change in distance between average nodal positions of tendon tissue measured with ultrasound speckle tracking and calcaneus marker positions from motion capture of healthy young adults walking at a speed of 1.25 m/s.

### Calculating Strain and Energy Storage

We determined subtendon strain in the longitudinal direction, which we called *longitudinal strain*, by dividing the change in length by the original length of the subtendon measured in the proximal-distal direction, consistent with *in vivo* methods of measuring AT strain (Kubo et al., 2002; Lichtwark and Wilson, 2005; Onambele et al., 2006). *Longitudinal strain* was equivalent to the proximal surface displacement divided by the initial distance between the distal and proximal cross sections (60 mm). We determined strain the fiber direction at the tissue-level, which we called *along-fiber strain*, by calculating the average along-fiber stretch (λ) in each subtendon and subtracting 1.

We calculated the energy stored in the tendon during loading by integrating the average tendon work relationship for the full AT (force in all subtendons versus the average subtendon length change), which we called *full tendon stored energy*. This approach was chosen to be consistent with *in vivo* methods of calculating AT negative work (Lichtwark and Wilson, 2005; Zelik and Franz, 2017). We alternatively calculated the energy stored in the tendon during loading by integrating the average tendon work relationship for the individual subtendons (force in individual subtendon versus that subtendon’s length change) then summing the work done by each subtendon; we called this value *summed-subtendon stored energy*. We also determined the total *strain energy* in the AT by integrating the strain energy density (Φ) across all the elements in the subtendon; the strain energy density is directly calculated using the tendon constitutive model.

Kruskal–Wallis tests were used to determine whether differences in *fascicle lengths* and *along-fiber strain* occurred between subtendons of the three models. *Post hoc* Wilcoxon rank sum tests were used to test for differences in subtendon *fascicle lengths* and *along-fiber strains* between each pair of models. The Holm–Bonferroni method was used to correct for family wise error rate for tests repeated over the three subtendons. Significance was set at *p* = 0.05.

## Results

### Undeformed Subtendon Fascicle Lengths Are Increasingly Longer Than Free Tendon Length at Greater Twist Angles

**FIGURE 2:**
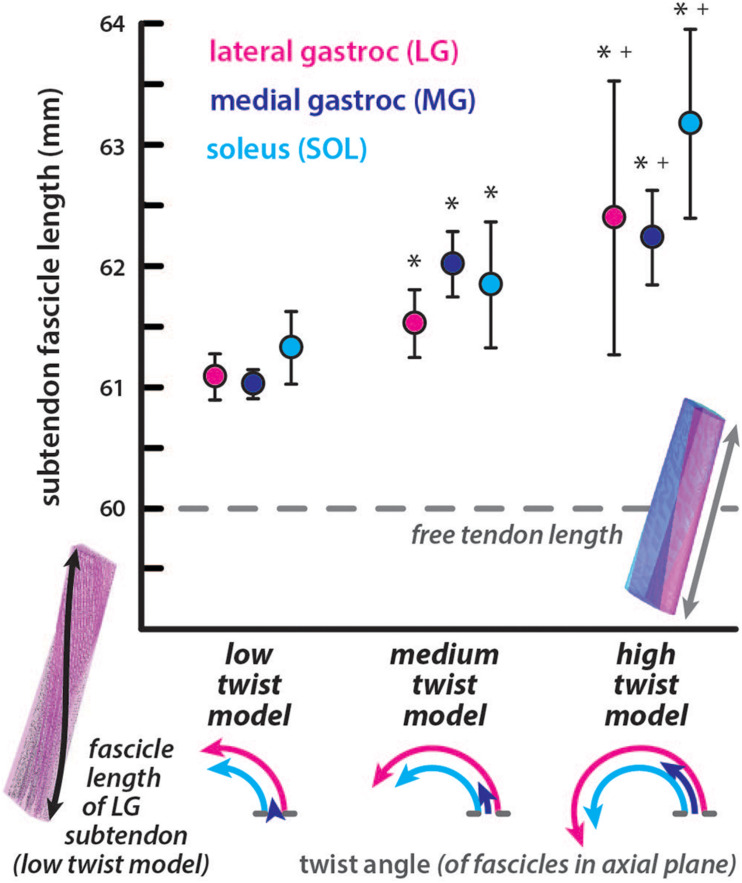
Subtendon *fascicle lengths* in the undeformed models are longer than resting free tendon length. *Fascicle lengths* are longer in subtendons of models with more twisted geometry. The average length of fascicle tracts in each subtendon are plotted with error bars indicating standard deviations. Significant differences (*p* < 0.00001) in lengths were found in subtendons compared to the low twist (^∗^) and medium twist (+) models. The dotted line indicates the length of the free tendon, which would be consistent with *fascicle lengths* if no twisting among or within the subtendons occurred. The *twist angle* of the fascicles within each subtendon are illustrated in the axial plane for each model.

All subtendon *fascicle lengths* were longer than the free tendon length of 60mm in the undeformed configuration (Figure 2). For the low twist model, subtendon *fascicle lengths* were: LG = 61.09 ± 0.19 mm, MG = 61.03 ± 0.12 mm, and SOL = 61.33 ± 0.30 mm. For the medium twist model, subtendon *fascicle lengths* were: LG = 61.53 ± 0.28 mm, MG = 62.02 ± 0.27 mm, and SOL = 61.85 ± 0.52 mm. For the high twist model, subtendon *fascicle lengths* were: LG = 62.39 ± 1.13 mm, MG = 62.24 ± 0.39 mm, and SOL = 63.18 ± 0.78 mm. The MG subtendon had the shortest fascicles and the SOL subtendon had the longest fascicles in low and high twist models. *Fascicle lengths* were more variable in subtendons with higher *twist angles*. For all subtendons, resting *fascicle lengths* were significantly shorter (*p* < 0.00001) in the low compared to the medium twist model, in the low compared to the high twist model, and in the medium compared to the high twist model. The percent differences in average *fascicle lengths* compared to the free tendon length were relatively small (low twist: LG = 1.80%, MG = 1.71%, and SOL = 2.19%; medium twist: LG = 2.52%, MG = 3.32%, and SOL = 3.04%; high twist: LG = 3.90%, MG = 3.66%, and SOL = 5.16%).

### Subtendon Along-Fiber Strains Are Lower Than Longitudinal Strain During Elongation and Are Lower With Greater Subtendon Twist

**FIGURE 3:**
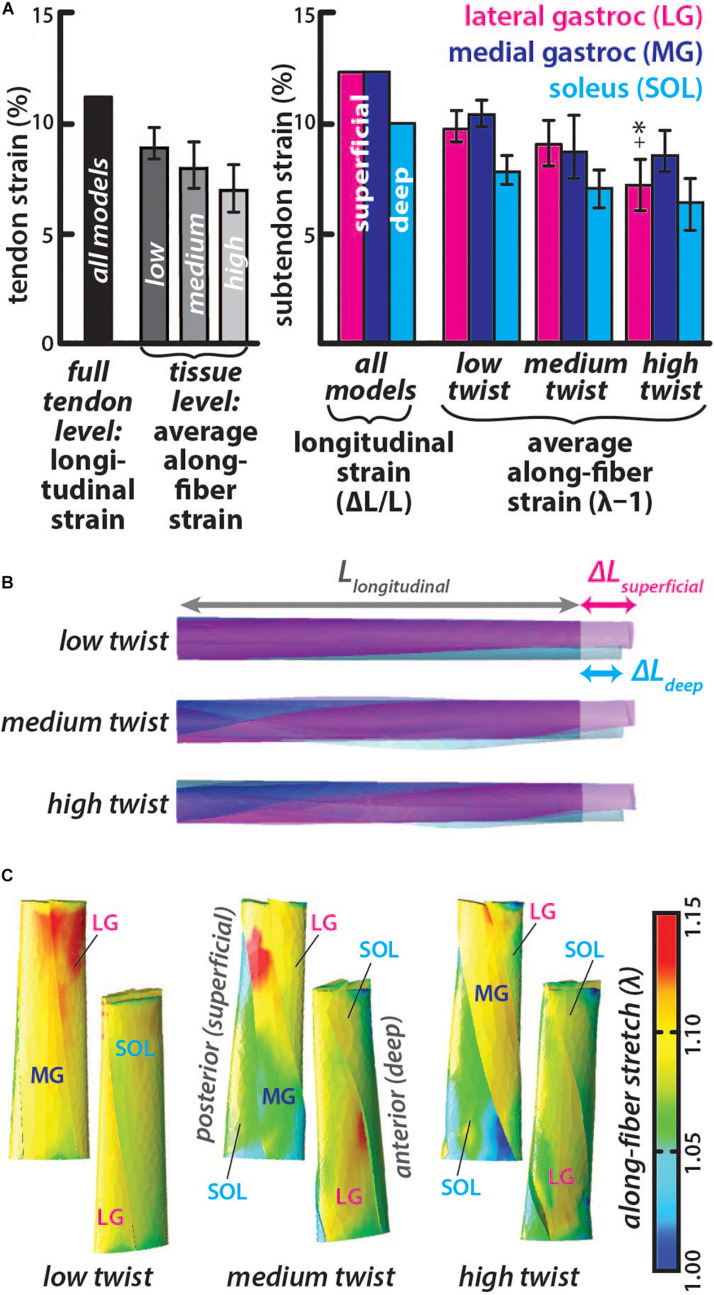
Subtendon *along-fiber strains* are lower than *longitudinal strain* and was lower in subtendons of models with greater twist. **(A)** The *longitudinal strain* was the same in all models and measured as the change in length in the proximal-distal direction divided by the initial length. The average *along-fiber strain* was determined in each full tendon model as well as in the individual subtendons, with error bars indicating standard deviations. Significant differences (*p* < 0.002) in *along-fiber strain* were found in high twist LG subtendon compared to the low twist (^∗^) and medium twist (+) models. **(B)** All twist models experienced the same elongation in the proximal-distal direction, with greater displacement in the superficial (at the proximal end) subtendons (LG and MG) than the deep subtendon (SOL). **(C)** Posterior and anterior view of each model show the along-fiber stretch (λ) from each simulation, where a stretch value of one is the zero-strain or undeformed condition.

The *longitudinal strain* was the same in all models with higher strain in the LG and MG subtendon (12.7%) than the SOL subtendon (9.8%) (Figures 3A,B), corresponding with differential displacement between the deep and superficial portions of the AT (Franz et al., 2015). Average *along-fiber strains* were lower than *longitudinal strains* in all models with values for each subtendon decreasing from the low twist model (LG = 9.9 ± 0.8%, MG = 10.5 ± 0.6%, and SOL = 7.9 ± 0.7%) to the medium twist model (LG = 9.2 ± 1.0%, MG = 9.0 ± 1.4%, and SOL = 7.1 ± 0.9%) to the high twist model (LG = 7.2 ± 1.2%, MG = 8.8 ± 0.9%, and SOL = 6.4 ± 1.2%), with significant differences (*p* < 0.002) in the LG of the low and medium twist models compared to high twist The MG subtendon had the highest average *along-fiber strain* in the low and high twist models and the SOL subtendon had the lowest average *along-fiber strain* in all three models (Figure 3A). *Along-fiber strains* were non-uniform throughout all subtendons in all of the models (Figure 3C). The percent differences in average *along-fiber strains* compared to the *longitudinal strains* ranged from 20-55% (low twist: LG = −25.7%, MG = −19.5%, and SOL = −22.5%; medium twist: LG = −33.0%, MG = −37.0%, and SOL = -32.3%; high twist: LG = −55.0%, MG = −38.6%, and SOL = −41.8%).

### Energy Stored in the Tendon for Similar Elongations Is Lower With Greater Subtendon Twist

**FIGURE 4:**
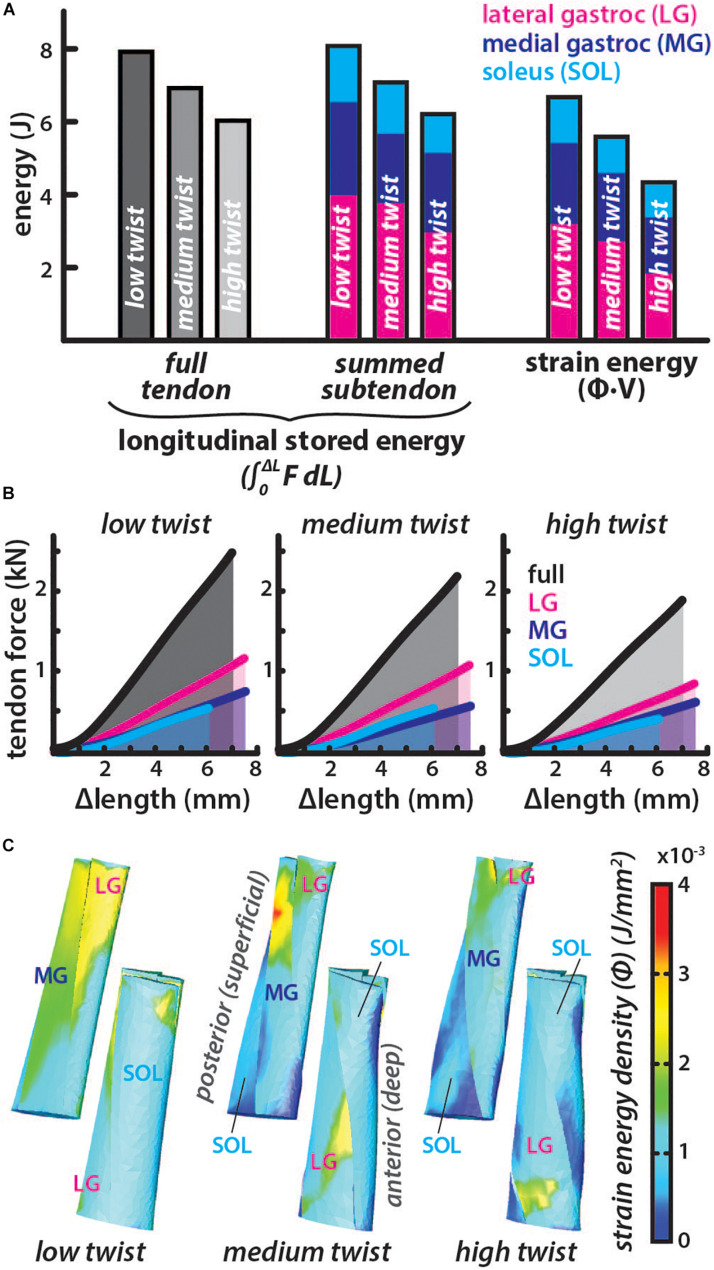
Energy stored in the tendon for similar elongations is lower in models with greater subtendon twist. **(A)** The total energy stored in each twist type was calculated longitudinally using two methods. *Full tendon stored energy* was calculated by integrating the force (F) from all subtendons over the average length change (ΔL) of the all subtendons. *Summed-subtendon stored energy* was calculated by integrating the force (F) from each subtendon over the average length change (ΔL) of that subtendon then summing the results. Total *strain energy* was also computed using the strain energy density (Φ) and volume (V) of each element. Colored bars show the proportion of energy stored in each subtendon. **(B)** The average tendon length change is plotted with the total force in black and the area under the curve was calculated to find *full tendon stored energy*. Subtendon length changes and forces are plotted with colored lines and area under each curve was summed to compute the *summed subtendon stored energy*. **(C)** Posterior and anterior view of each model show the strain energy density (Φ) from each simulation, which was multiplied with element volumes and summed to calculate total strain energy.

The average subtendon length change was the same in all models but the tendon force required to achieve the same displacement was lower in models with greater twist (low twist = 2.49 kN, medium twist = 2.18 kN, and high twist = 1.90 kN). Therefore, the *full tendon stored energy* was also lower in models with greater twist (low twist = 7.91 J, medium twist = 6.92 J, high twist = 6.04 J) (Figure 4). Similarly, the *summed-subtendon stored energy* was lower in models with greater twist (low twist = 8.10 J, medium twist = 7.09 J, and high twist = 6.22 J), though values were slightly higher than the *full tendon stored energy*. Total *strain energy* showed a similar trend as tendon stored energy and was lower in models with greater twist (low twist = 6.73 J, medium twist = 5.63 J, and high twist = 4.34 J), though *strain energy* in all models was less than the energy determined with both longitudinal methods. The strain energy density was non-uniform throughout all subtendons in all of the models, with areas of high energy corresponding with areas of high strain (Figure 3C). The percent differences in total *strain energy* from the *full tendon stored energy* increased with greater twist (low twist = −16.2%, medium twist = −20.5%, and high twist = −32.7%), while percent differences in total *strain energy* from the *summed-subtendon stored energy* displayed a similar trend and were slightly larger (low twist = −18.5%, medium twist = −23.0%, and high twist = −35.7%).

## Discussion

The primary goal of this study was to use models of the Achilles free tendon to explore how mechanical behavior varies with morphology differences in subtendon and fascicle twist. Models predicted that, with more twisted resting geometry, the AT had longer *fascicle lengths* when undeformed and exhibited reduced *along-fiber strain* and lower energies stored during elongation, thus altering the free tendon’s response to loading.

Models predicted that increasing the amount of twist of the Achilles subtendon resting morphology effectively lowered stiffness in the free tendon even though the tissue level material properties were held constant. In the models, the tissue-level strain varied with twist while the elongation was the same. The average *along-fiber strain* was lower in models with greater twist (Figure 3A), leading to larger differences in the strain at the tissue-level compared to the tendon-level strain. The distribution of strains within the tissue differed as well (Figure 3C). Similar to our results, AT FE models with twisted geometry developed by Shim et al. (2018) predicted that greater geometric twist redistributed internal stresses during loading allowing larger loads to be applied in simulations before stress in the tissue reached a given rupture limit. Tendon twisted geometry could be a mechanism to reduce the tendon tissue strains experienced during a given muscle-tendon-unit (MTU) excursion. A more twisted morphology may therefore aid in avoiding rupture as failure strain of tendon has been shown to be highly conserved between different tendons in different species (LaCroix et al., 2013).

In our simulations where tendon elongation was the same, greater twist corresponded with lower stored energy at both the tendon level (longitudinal stored energy) and at the tissue level (strain energy) (Figure 4A). Longitudinal energy differences can be attributed to the varied forces applied in each model for the same elongation (Figure 4B), while the *strain energy* differences were also influenced by variation in tissue-level strain (Figure 3A). Twisting may actually improve the efficiency of energy return by the tendon, as tendon-level strain and energy storage requires less tissue deformation that could lead to conformational changes at the collagen level that would result in energy loss. In fact, equine energy storing tendons with helical substructures have been shown to experience less hysteresis loss than postural tendons with less fascicle rotation (Thorpe et al., 2013). Furthermore, the lower forces required in higher twist tendon models to elongate as much as the low twist model (Figure 4B), suggest that the amount of twist would impact series-elasticity of the musculotendon unit. A more compliant AT may enable greater force control by the triceps surae muscles (Alexander, 2002) and has the potential to alter the efficiency of these muscles (Lichtwark and Wilson, 2007; Uchida et al., 2016).

It is unknown if the variation in morphologic twist between individuals is an adaptation to mechanical stimuli or simply a product of anatomical variability. However, the amount of twist seems to provide tradeoffs in injury prevention and energetic efficiency. The twist of the subtendons and the *twist angle* of their fascicles may be an important consideration in the repair of AT ruptures. As the amount of twist does not vary much between the right and left sides (Pękala et al., 2017), twist in the contralateral tendon could serve as a reference in reconstructing the ruptured tendon. Alternatively, twist could be applied before suturing in an effort to preserve compliance as elasticity decreases (Karatekin et al., 2018) or to protect the repaired tendon from further injury. This approach to improving AT reconstruction is an exciting opportunity for further studies.

Each of the three subtendons had different twist angle in all undeformed models, resulting in different average *fascicle lengths* (Figure 2). The MG subtendon had lower *twist angles* and generally shorter fascicles compared to the LG subtendon, and therefore experienced higher *along-fiber strains* even though *longitudinal strains* were the same in both subtendons (Figure 3). These results suggest that the MG subtendon is more vulnerable to injury due to its lower twist angle, which may help explain how failure of a single subtendon occurs, resulting in a partial tear in the Achilles (Smigielski, 2008). Further studies could work to determine if tears occur more often in the less twisted MG subtendon. The SOL subtendon had the greatest *twist angle* in all models and generally had longer fascicles. Subtendon twist within the free tendon influenced *fascicle lengths* in addition to the fascicle *twist angle*, so in the case of the medium twist model, the MG subtendon had longer fascicles despite its lower *twist angle*. The difference in *twist angles* between these subtendons could be associated with the diversity in morphology of the muscles they attach to. Dean et al. (2007) demonstrated that a twisted tendon in the jaw of a spotted ratfish facilitated more uniform strains in the fibers of a muscle with a broad attachment, so that these fibers could operate at similar lengths on the force-length curve. The triceps surae muscles also have broad attachments though the attachment morphology of each muscle is different (Dalmau-Pastor et al., 2014), possibly necessitating different subtendon twist angles to achieve the same equalizing effect on fiber operating lengths.

Our secondary goal was to assess how morphological complexity of the AT impacts how *in vivo* measurements of tendon behavior should be interpreted. We hypothesized that varying lengths and geometry resulting from twisted morphology may lead to errors during *in vivo* calculations of strain and energy. We found that resting *fascicle lengths* were only slightly underestimated by 1.5-5.5% by using measurements of tendon length. However, strains and energy storage measured at the fascicle level were both overestimated by 20–55 and 15–35%, respectively when using longitudinal methods that are consistent with *in vivo* measurements. The amount of error increases with twist. Unfortunately, definitively calculating measurement errors due to subtendon twist is not possible as methods of determining the amount of twist *in vivo* have not been developed, to our knowledge. Hopefully the future development of imaging techniques will allow for correction of such measurement errors in the future. Additionally, the high twist group accounts for less than 6% of the population studied in Pękala et al. (2017) so most tendons will likely have a low (48% of population) or medium (46% of population) amount of twist. Therefore, the extent of error associated with the low (strain = 19–26%, energy = 16–19%) and medium (strain = 32–37%, energy = 20–23%) twist models will represent most of the population included in *in vivo* studies.

There are several limitations to this study that should be noted. Our goal was to characterize the mechanical consequences of varying subtendon twist, independent of variation in tendon geometry or material properties. To this end we developed a model with a generic geometry so that we could create variations in internal structure and examine the effects on tendon behavior when loaded uniaxially. Future work to develop more detailed models is needed to fully investigate how subtendon twist influences *in vivo* AT behavior. Methods enabling *in vivo* quantification of subtendon twist would improve subject-specific models. Twist could be incorporated with other model inputs, like geometry and material properties, that tendon behavior is sensitive to Shim et al. (2014). Furthermore, validation with *in vivo* data is required to better understand the predictions made in this study. We chose to use the measurements of subtendon structure reported by Pękala et al. (2017) because of the extent of quantitative anatomical data provided. We are aware that the subtendon proportions and cross section in that study (Pękala et al., 2017), deviate somewhat from those reported by previous authors (Szaro et al., 2009; Edama et al., 2015). Since measurements in these studies were performed *ex vivo* in dissected tendons, a major assumption of our model was that subtendon twist was a morphological characteristic that could exist independent of loading. Therefore, we implemented twist in the geometry of the undeformed models, as opposed to applying a torsional loading condition to achieve twist. AT rotation has been observed in response to *in vivo* loading (Obst et al., 2014), and model twist likely changes during simulated loading. All model *twist angles* are reported for the undeformed configuration.

All tendon measurements used to create model geometries were made in fresh frozen cadavers (Pękala et al., 2017), though simulation conditions were chosen to represent *in vivo* tendon loading (Franz et al., 2015). Displacements of subtendon proximal ends were controlled to match elongations estimated with speckle tracking in the free tendon. In the *in vivo* experiments, elongation was calculated from the proximal end to the calcaneal marker. The distal end of the models was located at the superior edge of the calcaneus, which resulted in shorter model tendons and therefore higher strains than in the *in vivo* study. Although AT strains of this magnitude have been reported during one-legged hopping (Lichtwark and Wilson, 2005), model predicted strains are likely larger than what occurs during walking. However, all models experienced the same *longitudinal strains*, allowing for comparison of tissue level strains due to differences in subtendon twist. Further, conclusions about strain in each subtendon are difficult to interpret from these results as the free tendon modeled here does not capture full external portion of proximal tendon associated with lateral or MG head. Simulations were performed quasi-statically and only included tendon loading. Further work is needed to incorporate more detailed viscoelastic behavior of AT in order to accurately simulate full tendon work loops. We assumed the tendon was unloaded prior to simulated elongation and, thus, we applied no initial stretch to the models. Estimates of *in vivo* AT loading show that the tendon is not stress-free at the beginning of the gait cycle (Keuler et al., 2019). As the start of our simulations correspond to this time point, it may be appropriate to apply a pre-stretch to the subtendons, which would affect when the tendon would transition from the toe region to the linear portion of the stress-strain curve, leading to higher stresses at the strains enforced in this study. While estimates of stress in the full tendon exist, it is unclear how loading may be distributed between the subtendons. Evidence of differences in slack angles of the triceps surae muscles (Hirata et al., 2015) suggests that the initial stretch at a given joint angle would vary between the subtendons, though current methods are unable to estimate what these loads should be. An exciting direction for future work would be to investigate how inhomogeneous subtendon loading in addition to variation in subtendon morphology influence the behavior of the AT.

In conclusion, the models developed in this paper of the AT with varied subtendon twisted geometry help us understand how this morphological characteristic can result in different amounts of tissue strain and energy storage within the tendon in response to similar loading. High *twist angles* in tendon fascicles can contribute to errors in quantifying these mechanical behaviors when methods that rely on 2D measurements at the endpoints are employed. The knowledge of this effect will aid in the interpretation of future studies of AT behavior and inspire future work to design methods that enable measurements of *in vivo* subtendon structure.

## Data Availability Statement

The datasets generated for this study are available on request to the corresponding author.

## Author Contributions

KK and SB: conceptualization, writing – review, and editing. KK: model development and analysis and writing – original draft preparation. SB: funding acquisition. Both authors contributed to the article and approved the submitted version.

## Conflict of Interest

The authors declare that the research was conducted in the absence of any commercial or financial relationships that could be construed as a potential conflict of interest.
